# Translation, Cross-Cultural Adaptation, and Psychometric Validation of the Positive Body Image among Adolescents Scale (PBIAS) into Spanish and Catalan

**DOI:** 10.3390/ijerph20054017

**Published:** 2023-02-23

**Authors:** Glòria Tort-Nasarre, Eva Artigues-Barberà, Mercè Pollina-Pocallet, Anna Espart, Judith Roca, Josep Vidal-Alaball

**Affiliations:** 1 SAP ANOIA, Gerència Territorial Catalunya Central, Institut Català de la Salut (ICS), 08700 Igualada, Spain; 2 Department of Nursing and Physiotherapy, Faculty of Nursing and Physiotherapy, University of Lleida, 25198 Lleida, Spain; 3 AFIN, Research Group and Outreach Centre, Universitat Autònoma de Barcelona, 08193 Bellaterra, Spain; 4 Balàfia Primary Care Center, Gerència Territorial Lleida, Institut Català de la Salut (ICS), 25005 Lleida, Spain; 5 Fundació Institut Universitari per a la Recerca a l’Atenció Primària de Salut Jordi Gol i Gurina (IDIAPJGol), 08007 Barcelona, Spain; 6 Bellpuig Primary Care Center, Gerència Territorial Lleida, Institut Català de la Salut (ICS), 25250 Lleida, Spain; 7 Development of Healthy and Sustentable Organizations and Territories (DOTSS), Serra Húnter Lecturer, 25001 Lleida, Spain; 8 Research Group of Health Care (GRECS), Lleida Institute for Biomedical Research, Dr. Pifarré Foundation, IRBLleida, 25198 Lleida, Spain; 9 Health Promotion in Rural Areas Research Group, Gerència Territorial de la Catalunya Central, Institut Català de la Salut (ICS), 08272 Sant Fruitós del Bages, Spain; 10 Department of Medicine, University of Vic-Central University of Catalonia, 08500 Vic, Spain

**Keywords:** body image, adolescents, adolescent mental health, psychometrics, instrument validation, cross-cultural adaptation

## Abstract

The Positive Body Image among Adolescents Scale (PBIAS) explores the factors that bolster and interfere with developing and maintaining a positive body image during adolescence. The aim of this study was to translate, adapt, and validate the PBIAS into Spanish and Catalan. A cross-sectional study was conducted for the instrument’s translation, cross-cultural adaptation, and psychometric validation. A process of translation, back-translation, expert consultation, and piloting was followed. The reliability and statistical validity were evaluated. The Cronbach’s alpha was 0.95 in both the Spanish and Catalan versions. Pearson’s correlation coefficients were statistically significant (r > 0.087) for all items analyzed. The resulting values of the Spanish and Catalan versions indicate a good level of concordance (*p* < 0.001) with the original questionnaire, the comparative fit index being 0.914 and 0.913, the Tucker–Lewis index being 0.893 and 0.892, the root mean square error of approximation being 1.31 and 1.28, and the standardized root mean square residual being 0.051 and 0.060, respectively. The instrument presents a good level of internal consistency, a high level of reliability, and statistical validity compared to the original instrument. The PBIAS in Spanish and Catalan can be a useful assessment instrument for educators and health professionals in the context of adolescent mental health literacy. This work contributes to the Sustainable Development Goals (Goal 3) of the United Nations 2030 Agenda.

## 1. Introduction

Body image is a multifaceted construct that encompasses affective, cognitive, perceptual, and behavioral aspects related to one’s body and appearance [1]. The body image construct has multiple meanings among adolescents and a mismatch between actual and desired body image does not necessarily imply dissatisfaction [2]. However, several health correlates and consequences of having a negative body image, e.g., depression, anxiety, low self-esteem, substance abuse, unhealthy weight control practices, and disordered eating [3,4,5,6], and a positive body image, e.g., health-promoting behaviors and greater well-being [7,8], have been established.

Adolescence is a period of both physical and emotional change that involves an adjustment of the perception of the person as an independent individual who makes his or her own decisions. The different developmental processes that characterize adolescence could affect the positive experiences of one’s body image [9]. Sometimes, experiences and self-perception can lead to emotional distortions that affect mental health. A maladjusted perception of body self-image may contribute to the development of significant mental health problems [10]. During adolescence, this perception is closely related to body weight and gender [11,12]. 

Therefore, the formation of positive body attitudes and perceptions during adolescence would be considered essential. In recent years, research focused on positive body image and its links to well-being has been carried out [13]. According to Tylka and Wood-Barcalow, a positive body image is defined as an individual’s love, respect, acceptance, and appreciation of and comfort with their body, regardless of their actual physical appearance, as well as their ability to interpret messages in a body-protective manner [14]. However, this conceptualization was generated from adult research findings and does not include factors that are unique to the developmental context of adolescents [15].

During the process of change and acceptance of one’s own body image, which involves the transition from childhood to adolescence and then to adulthood, school nurses play a key role. The early detection of disturbances in mental well-being, which often accompany disorders in the acceptance of self-image, are often identified for the first time by educators and health professionals, such as school nurses [16,17]. Adolescent mental health literacy, as a part of health education, is a critical element for health professionals such as school nurses working with this population group [18], as well as other health professionals such as psychologists or pediatricians. Although there are tools to assess emotional and mental well-being in adolescents, the range of tools available to assess body image and emotional well-being specifically in adolescents and in different languages is not particularly extensive. Often these tools are designed for culturally and linguistically different populations rather than those we wish to assess. 

Some of the instruments used to assess positive body image among adults cited in Maes’ study [9] are, for example, scales based on self-care such as the Body Responsiveness Questionnaire (BRQ) [19] or the Mindful Self-Care Scale (MSCS) [20]; scales based on the appreciation of the body such as the Body Appreciation Scale-2 (BAS-2) [21]; or scales based on self-acceptance, such as the Body Image Coping Strategies Inventory (BCSI) [22]. 

In this regard, the Positive Body Image among Adolescents Scale (PBIAS) developed by Maes et al. measures the construct of a positive body image among adolescents. The PBIAS is a questionnaire validated in 2021, consisting of fifteen items grouped into four factors. Factor 1 includes questions about the adolescent’s own body appreciation; Factor 2 includes questions about others’ body esteem; Factor 3 includes questions about the adolescent’s resilience to the body’s ideal appreciation in the media; and Factor 4 includes questions about the adolescent’s resilience to negative comments about their appearance. Each dimension shares similarities with conceptualizations based on body image during adulthood but is described more in the context of adolescent development. In addition, the PBIAS is different in the sense that it encompasses, in a single scale, components that had been studied separately, with the following four subscales: appreciation of one’s own body, appreciation of other people’s bodies, resilience to body ideals in the media, and resilience to negative feedback on appearance [9]. 

The PBIAS may be useful for a significant group of professionals and for researchers who wish to explore the factors that may bolster and interfere with developing and maintaining a positive body image during adolescence [9]. Similarly, it may be useful for clinicians and educators in their endeavors to help adolescents develop and maintain a positive body image. 

Thus, the main objective of the study was to describe the process of translating, culturally adapting, and validating the PBIAS in order to assess a positive body image in the Spanish and Catalan context. A secondary objective is to provide a new tool adapted to Spanish- or Catalan-speaking educators and health professionals to assess body image disturbances in adolescents.

## 2. Materials and Methods

### 2.1. Study Design

The design of the study was carried out in two phases. The first phase involved translation and cultural adaptation, a process that required verification of the metric properties of the Spanish and Catalan languages. In the second phase, a cross-sectional analytical design was used to analyse the cross-cultural adaptation, validation, and reliability assessment of the instrument, using Cronbach’s alpha for internal consistency and confirmatory factor analysis (CFA) to assess factorial validity. 

### 2.2. Participants

A convenience sample of 222 participants was selected from a reference population of adolescents. The adolescents were selected from three secondary schools in Catalonia (Spain) in the cities of Capellades, Calaf, and Lleida.

The sample size was based on a person–item ratio of at least 10 subjects per item in the instrument for general psychometric approaches: scale and item analysis, Pearson’s correlations, and exploratory factor analysis [23]. The inclusion criteria were being a secondary school student, being aged between 12 and 18 years, and voluntary participation. No exclusion criteria other than not meeting the eligibility criteria were considered.

### 2.3. Variables and Procedures

First, permission to translate the PBIAS into Spanish and Catalan and then culturally adapt and validate its content was obtained from the main author of the original instrument [9]. As the original, the scale has fifteen Likert-type items with seven possible response options: (1) completely disagree; (2) strongly disagree; (3) rather disagree; (4) neither agree nor disagree; (5) rather agree; (6) strongly agree; and (7) completely agree. 

The PBIAS was tested on Belgium adolescents for validity and reliability with strong consistency (with comparative fit index (CIF) = 0.913, Tucker–Lewis index (TLI) = 0.892, root mean square error of approximation (RMSEA) = 1.28, and standardized root mean square residual (SRMR) = 0.060), and was translated from Dutch into English to provide an option for English-speaking countries [9]. 

The English version of the questionnaire was translated into Spanish and Catalan and adapted to the cultural context. To ensure the credibility of the translation, we have tried to keep as close as possible to the original English text. However, a direct translation was not possible because some words had to be changed to match the Spanish and Catalan language and context. The recommendations for a transcription process outlined by the World Health Organization [24] and the recommendations of Sousa et al. [23] were followed. Two translators, bilingual in Spanish and Catalan and proficient in English, one of them an expert in health terminology and the other in linguistics, participated independently in the translation and back-translation. Two expert reviewers and three adolescents were also asked to verify the clarity and comprehension of the instrument. Based on suggestions to improve its comprehensibility, the instrument was subsequently piloted on 20 adolescents, who did not suggest any changes. Once this process was completed, the final version was approved.

Sociodemographic data were provided for the study: school grade (1st to 4th secondary education and ESO in the Spanish educational system), age (12 to 18 years old), gender (female, male, and non-binary), weight (kg), height, (cm), and whether they had suffered or were suffering from any eating disorder (yes, no, and I am not sure).

### 2.4. Data Collection

Data were collected between May and June 2022. The questionnaire used in this study was created online with Microsoft Forms^®^ (Microsoft Corporation, Redmond, WA, USA). The questionnaire link (https://www.microsoft.com/en-us/microsoft-365/online-surveys-polls-quizz, accessed on 7 January 2023) was sent to the school’s teaching coordinator, who forwarded it to the students through the school’s internal platform. Students answered the questionnaire during a normal lesson to ensure high participation. Students were asked to complete the questionnaire first in Spanish and then, without leaving the online platform, to complete the same questionnaire in Catalan. The estimated time to answer the entire questionnaire was 15 min. Responses were also received through Microsoft Forms^®^.

### 2.5. Ethical Considerations

The study was approved by the University Institute for Research in Primary Care (IDIAP) Jordi Gol i Gurina ethics committee (Code 21/295-P). Consent was requested from participants over 15 years of age and, from minors, consent was requested from their mother, father, or legal guardian, in compliance with the European Data Protection Regulation 2016/679 of the European Parliament and of the Council of 27 April 2016, and the Organic Law 3/2018 of 5 December, on the protection of personal data and guarantee of digital rights. Data confidentiality and anonymity were ensured throughout the process by assigning each participant an alpha-numeric code. 

### 2.6. Data Analysis

A descriptive analysis was performed to characterize the participants in the study. Continuous data were described by means and standard deviations (i.e., years, weight, and height); while categorical data were described as frequencies and percentages (i.e., years, grade, gender, and eating disorder status). After this analysis, a set of tests was carried out to assess the internal consistency and validity, and to confirm the best-structured model of factor dimensions.

Following a similar methodology used by other authors [25,26], the internal consistency was measured through a Cronbach’s alpha analysis, and CFA was performed to assess factor validity. A principal component analysis (PCA) was carried out to determine the factor structure of the instrument in the cross-cultural version in Spanish and Catalan. For this purpose, varimax was performed as a rotation method, Bartlett’s test of sphericity was used to check for assumptions, and the Kaiser–Meyer–Olkin (KMO) test was performed for sampling adequacy. To analyze construct validity, eigenvalues > 1 were identified and Pearson’s correlation coefficient was calculated for each scale item with the whole scale. To analyze the best model structure, fit indices were compared using CFI, TLI, RMSEA, and SRMR. The confidence level used was 95%. Jamovi version 2.2.5 (The jamovi project 2021, Sydney, Australia) was used to conduct the statistical analyses of the study.

## 3. Results

A total of 222 students between 12 and 18 years old participated in the study (x̄ = 15.2, SD = 1.01, and mean = 15). The largest groups in the study were participants between 15 and 17 years old (72.1%), female (56.8%), and those not related to eating disorders (71.5%) (Table 1).

The overall value of internal consistency, measured using Cronbach’s alpha, was 0.951 in both the Spanish and Catalan versions, indicating a good level of internal consistency for all 15 items included in the questionnaire.

The four-factor dimensions of the original instrument (i.e., appreciation of one’s own body, appreciation of other people’s bodies, resilience to body ideals in the media, and resilience to negative feedback on appearance) were also analyzed in these versions through CFA. The resulting values of the Spanish and Catalan versions indicate a good level of concordance (*p* < 0.001) with the English questionnaire, being the CFI 0.914 and 0.913, the TLI 0.893 and 0.892, the RMSEA 1.31 and 1.28, and the SRMR 0.051 and 0.060, respectively.

Despite the values obtained for the four-factor dimensions, a PCA was conducted to find out whether a different factor dimension structure resulted in a better model. The overall KMO values were 0.917 in the Spanish version and 0.91 in the Catalan version; the Bartlett’s test of sphericity result was the same for both instruments, df = 105 and *p* < 0.001. Based on an eigenvalue greater than one, the best factor dimensions fit with three-factor dimensions in both versions (Table 2). The three factors explained the 80.7% and 78.7% of the cumulative variance in the Spanish and Catalan instruments, respectively (Table 3).

In the analysis of construct validity, using Pearson’s correlation coefficient for each of the items with the whole scale, the significant values (r > 0.087; CI = 95%) were obtained for each of the items in both versions (Table 4).

A new CFA was conducted including only three-factor dimensions (i.e., appreciation of one’s own body, appreciation of other people’s bodies, and resilience to body ideals in the media and negative feedback on appearance). The resulting values indicated a significant (*p* < 0.001) but slightly poorer level of concordance compared with the four-factor dimensions regarding the CFI, TLI, RMSEA, and SRMR (Table 5).

## 4. Discussion

The aim of the study was to describe the process of translating the PBIAS into Spanish and Catalan and then perform a cross-cultural adaptation and validation of both versions. After translation according to the main standards [23], the results of the statistical tests showed that the internal consistency of the instrument calculated with Cronbach’s alpha analysis was analogous (α = 0.951) to the original instrument [9].

The PCA measured in our work was used to assess the cross-cultural adaptation of the scales and indicated that, unlike in the original instrument [9], a possible structured model of three factorial dimensions could also fit as a good model in the Spanish and Catalan versions. In this new three-factor model, the statements included in the original factor ‘Resistance to media body ideals’ also seemed to fit into the factor ‘Resistance to negative opinions about appearance’. In this sense, items 1 to 6 of the scale would be included in factor 1 (body self-esteem), items 7 to 9 would be included in factor 2 (others’ opinions about my body), and items 10 to 15 would be included in factor 3 (resistance to negative body image ideals and feedback). However, the analysis comparing the model fit indices did not show the same result as the PCA. Despite the fact that values obtained in the comparison of the four- versus three-factor dimension model were not too different, in all indices the results were better in the four-factor dimension model, based on the fact that a good model is defined by the following values: CFI > 0.95, TLI > 0.95, RMSEA = 0.05 (or 0.08 as an acceptable model), and SRMS < 0.10.

These results confirm that although cultural adaptation allows the generation of a three-factor model, the similar results between the three- and four-factor models in both cases could be comparable to the four-factor model of the original version. In this study, the exploratory factor analysis (EFA) result was not calculated since the fifteen items of the original questionnaire already indicated four factors as the best-structured model. The authors concluded that it would be more relevant to conduct a PCA to ascertain whether a new factor dimension model should be used [9]. Although the possibility of using a new model was presented, it turned out not to be as adequate as the four-factor dimension model. The achieved results in the analytical tests allowed us to choose a four-factor dimension model as the best-structured model for adapting and validating the Spanish and Catalan versions of the PBIAS.

So far, the PBIAS scale has only been validated in Dutch and English [9] and in Spanish and Catalan. This may be due to the short time since the scale was first validated. It is likely that in time, as has happened with other scales [27,28,29,30], there will be further translations, adaptations, and validations of this scale into other languages and cultures, which will allow further studies to be carried out and it will become clearer whether the best model continues to be the four-factor model or whether there are other studies that also find the three-factor model to be valid. 

Thanks to this study, the Spanish version of the PBIAS is now available. This not only guarantees the existence of a positive body image assessment instrument for adolescents in Spanish-speaking countries (i.e., Spain and Latin America), but, together with the English version, the PBIAS can be used in countries where both languages coexist in parallel, such as in the United States. The validation of this scale in Spanish and Catalan increases the number of tools [31] in these languages that educators and health professionals can use in the prevention, detection, and assessment of mental health disorders in adolescents related to body image. More specifically, the PBIAS in Spanish and Catalan will make it possible to work on an important aspect such as the perception that many adolescents have of their own bodies and that of their peers. Through this scale, educators and health professionals will also be able to create working materials that are adapted to the needs of this type of adolescent and address mental health problems in a more specific and targeted way in high schools [32].

The validation of this tool in these two languages not only contributes to the achievement of the project’s objectives but also to target 3.4 of the Sustainable Development Goal 3 (ensure healthy lives and promote well-being for all at all ages) [33]. 

### Limitations

This study has identified three main types of constraints. The first is that we did not use a random sample, but a convenience sample. Another limitation is the heterogeneity of the participants in terms of gender (56.8% females versus 39.6% males) and age groups (26.4% were 12 to 14 years old, 72.1% were 15 to 17 years old, and 1.8% were older than 17 years old). This could lead to a potential selection bias affecting the obtained results. Finally, it was considered a limitation that the participants were not asked if they were taking any psychotropic drugs or if they were suffering from any mental disturbances at the time of the study that might influence their responses in the questionnaires.

Future studies are needed to confirm the results presented here and to determine the influence of these limitations and possible biases introduced in this study.

## 5. Conclusions

The Spanish and Catalan versions of the PBIAS instrument showed high levels of reliability and statistical validity comparable with the original version in English, and, therefore, it is a suitable instrument for assessing positive body image among adolescents in the Spanish and Catalan context. Educators and health professionals can implement this scale in their work with adolescents to detect potential mental health problems. This work provides a tool that contributes to the achievement of Goal 3 of the Sustainable Development Goals.

## Figures and Tables

**Table 1:** Characteristics of the participants.

Total Participants (N = 222)			
		**Mean**	**SD**
Age (years old)		15.2	1.01
Weight (kg)		59.6	12.9
Height (cm)		166	14.3
**Frequencies**		**n**	**%**
Age (years)	12–14	58	26.4
	15–17	158	72.1
	>17	4	1.8
School grade	First ESO	3	1.35
	Second ESO	55	24.7
	Third ESO	85	38.2
	Fourth ESO	79	35.5
Gender	Female	126	56.8
	Male	88	39.6
	Non-binary	8	3.6
Are you suffering/have you ever suffered from an eating disorder (e.g., anorexia or bulimia)?	Yes	38	17.2
	No	158	71.5
	I am not sure	25	11.3

SD: standard deviation. ESO: compulsory secondary education in the Spanish educational system (*Educación Secundaria Obligatoria*).

**Table 2:** Principal Component Analysis of the Spanish and the Catalan versions.

		Spanish Version			Catalan Version		
		Components					
		1	2	3	1	2	3
1.	I feel content with the way my body looks.	0.852			0.864		
2.	I love my body.	0.880			0.888		
3.	I accept all different features of my body, they make me who I am (e.g., my eyes).	0.785			0.791		
4.	I feel content with the way my body has changed/is changing during puberty (e.g., my breasts have grown or my penis has grown).	0.791			0.759		
5.	I respect my body.	0.775			0.751		
6.	My body, what it looks like, and what it can do are all part of who I am as a person.	0.662			0.690		
7.	It is my hope that all teenagers are able to feel good about the changes that happen in their bodies during puberty.			0.896			0.881
8.	It is my hope that everyone is able to love their bodies as they are.			0.903			0.907
9.	It is my hope that everyone is able to view their bodies as a unique part of who they are as a person.			0.890			0.876
10.	If I am confronted with body ideals (e.g., slim or muscular bodies) in the media, I (would) try to distract myself by other things I like about the media.		0.780			0.751	
11.	If I am confronted with body ideals (e.g., slim or muscular bodies) in the media, I (would) try to distract myself and think about something positive.		0.781			0.799	
12.	If I (would) receive negative feedback on my appearance (e.g., from friends), it is important not to pay too much attention to this.		0.771			0.706	
13.	If I (would) receive negative feedback on my appearance (e.g., from friends), I (would) try to forget about this.		0.767			0.694	
14.	If I (would) receive negative feedback on my appearance (e.g., from friends), I (would) remind myself that those remarks are not important and that I love my body.		0.658			0.638	
15.	If I (would) receive negative feedback on my appearance (e.g., from friends), I (would) try to distract myself and think about something positive.		0.750			0.741	

Rotation method: varimax. Assumption checks: Bartlett’s tests of sphericity and Kaiser-Meyer-Olkin (KMO) measure of sample adequacy. Number of components: based on eigenvalue >1.

**Table 3:** Eigenvalues and cumulative variance of items (only shown for eigenvalues >1).

	Spanish Version		Catalan Version	
	Eigenvalue	Cumulative Variance (%)	Eigenvalue	Cumulative Variance (%)
1	9.1376	60.9	8.875	59.2
2	1.7221	72.4	1.609	69.9
3	1.2402	80.7	1.321	78.7

**Table 4:** Pearson’s correlation coefficients of items with whole scale score.

Pearson’s Correlation of Items with Total Scale Score				
Item	Spanish Version		Catalan Version	
	r	*p* Value	r	*p* Value
1	0.809	<0.001	0.792	<0.001
2	0.830	<0.001	0.817	<0.001
3	0.847	<0.001	0.827	<0.001
4	0.808	<0.001	0.792	<0.001
5	0.877	<0.001	0.861	<0.001
6	0.827	<0.001	0.837	<0.001
7	0.714	<0.001	0.743	<0.001
8	0.693	<0.001	0.712	<0.001
9	0.634	<0.001	0.683	<0.001
10	0.702	<0.001	0.659	<0.001
11	0.747	<0.001	0.693	<0.001
12	0.788	<0.001	0.771	<0.001
13	0.761	<0.001	0.737	<0.001
14	0.807	<0.001	0.810	<0.001
15	0.820	<0.001	0.764	<0.001
**Pearson’s Correlation Coefficients**				
**Item**	**Spanish Version**		**Catalan Version**	
	**r**	***p* Value**	**r**	***p* Value**
1	0.809	**<0.001**	0.792	**<0.001**
2	0.830	**<0.001**	0.817	**<0.001**
3	0.847	**<0.001**	0.827	**<0.001**
4	0.808	**<0.001**	0.792	**<0.001**
5	0.877	**<0.001**	0.861	**<0.001**
6	0.827	**<0.001**	0.837	**<0.001**
7	0.714	**<0.001**	0.743	**<0.001**
8	0.693	**<0.001**	0.712	**<0.001**
9	0.634	**<0.001**	0.683	**<0.001**
10	0.702	**<0.001**	0.659	**<0.001**
11	0.747	**<0.001**	0.693	**<0.001**
12	0.788	**<0.001**	0.771	**<0.001**
13	0.761	**<0.001**	0.737	**<0.001**
14	0.807	**<0.001**	0.810	**<0.001**
15	0.820	**<0.001**	0.764	**<0.001**

r: Pearson’s correlation coefficient. In bold: statistically significant *p* value.

**Table 5:** Comparison of fit indices to determine the best model structure.

	Spanish Version (Four-Factors)	Spanish Version (Three-Factors)	Catalan Version (Four-Factors)	Catalan Version (Three-Factors)
CFI	0.914	0.870	0.913	0.866
TLI	0.893	0.843	0.892	0.838
RMSEA	0.131	0.158	0.128	0.156
SRMR	0.051	0.062	0.060	0.075

CFI: comparative fit index; TLI: Tucker–Lewis index; RMSEA: root mean square error of approximation; and SRMR: standardized root means square residual.

## Data Availability

Please contact the corresponding author (anna.espart@udl.cat) to request data.
